# The Location of the Fibular Tunnel for Anatomically Accurate Reconstruction of the Lateral Ankle Ligament: A Cadaveric Study

**DOI:** 10.1155/2021/5575524

**Published:** 2021-03-18

**Authors:** Jeong-Hyun Park, Hyung-Wook Kwon, Digud Kim, Kwang-Rak Park, Mijeong Lee, Yu-Jin Choi, Jaeho Cho

**Affiliations:** ^1^Department of Anatomy & Cell Biology, Graduate School of Medicine, Kangwon National University, Kangwon, Republic of Korea; ^2^Department of Orthopaedic Surgery, Chuncheon Sacred Heart Hospital, Hallym University, Chuncheon, Republic of Korea

## Abstract

We aimed to describe the location of fibular footprint of each anterior talofibular ligament (ATFL) and calcaneofibular ligament (CFL), as well as their common origin in relation to bony landmarks of the fibula in order to determine the location of the fibular tunnel. In 105 ankle specimens, the center of the footprints of the ATFL and CFL (cATFL and cCFL, respectively) and the intersection point of their origin (intATFL-CFL) were investigated, and the distances from selected bony landmarks (the articular tip (AT) and the inferior tip (IT) of the fibula) were measured. Forty-two (40%) specimens had single-bundle ATFL, and 63 (60%) had double-bundle patterns. The distance between intATFL-CFL and IT was 12.0 ± 2.5 mm, and a significant difference was observed between the two groups (*p* = 0.001). Moreover, the ratio of the intATFL-CFL location based on the anterior fibular border for all cadavers was 0.386. The present study suggests a reference ratio that can help surgeons locate the fibular tunnel for a more anatomically accurate reconstruction of the lateral ankle ligament. Also, it may be necessary to make a difference in the location of the fibular tunnel according to the number of ATFL bundles during surgery.

## 1. Introduction

Ankle sprain is the most common sports-related injury that usually involves the lateral ligament complex (LLC) of the ankle [1, 2]. Among the three ligaments of the LLC, anterior talofibular ligament (ATFL) rupture occurs in 80% of patients, the rest showing rupture of the calcaneofibular ligament (CFL) combined with ATFL. The latter condition causes instability of the ankle or subtalar joint, although the posterior talofibular ligament (PTFL) is rarely involved [3–6]. Nonoperative treatment is generally performed for acute ankle sprain, but failed conservative treatment causes chronic ankle instability (CAI) that may require surgical treatment; it has an incidence of ~10-30% of patients [3, 7–9].

To achieve a good clinical result for CAI treatment, many surgical techniques with anatomical repair or reconstruction of the ATFL and/or CFL have been proposed [10–14]. Early techniques for repair or reconstruction of the ATFL and/or CFL were invasive and nonanatomic. Common complications of this procedure include delayed recovery, wound infection, and nerve damage [15, 16]. The minimally invasive surgical (MIS) technique recently emerged and reduces the incidence of postoperative complications. Also, these procedures using percutaneous or arthroscopic techniques are focused on the anatomic repair or reconstruction of the ATFL and/or CFL [17].

The fibular origins of ATFL and CFL are concentrated in the lower part of the lateral malleolus and connected with connective fiber [18]. Also, ligament injury is likely to occur at the fibular side or substantial part of the ligament [4, 6]. Most MIS procedures for CAI require a single fibular tunnel with a common origin site of ATFL and CFL in both percutaneous and arthroscopic techniques [7, 9, 17, 19, 20]. Therefore, the identification of the common origin site of the ATFL and CFL for precise construction of the fibular tunnel is essential to reconstruct the lateral ankle ligament more anatomically.

Therefore, we aimed to describe the location of the fibular footprint of each ATFL and CFL, as well as the location of their common origin in relation to the easily identifiable bony landmarks of the fibula. Furthermore, we intend to suggest a reference ratio that can easily detect the location of the fibular tunnel as the common origin site of the ATFL and CFL by considering the anatomical variation of the ligaments.

## 2. Materials and Methods

This study was approved by the Institutional Ethics Committee.

105 specimens were included for this study. Of the 105 ankle specimens dissected from adult formalin-fixed cadavers, 40 (38.1%) were from females and 65 (61.9%) from males. The mean age of the donors at death was 76.4 (range, 44-99) years. Subjects with traces or scars from trauma or surgery on the skin and those with bony morphologic deformation due to bone union after fracture in the lateral aspect of the ankle were excluded. In addition, in order to discriminate the history of ligament injury, subjects in whom the shape of the ligament itself was damaged by previous injury after exposing the ligament through dissection were excluded. Even if the shape of the ligament itself was preserved, subjects in whom the tension of the ligament was not maintained and the fiber of ligament was abnormal when the ankle joint was placed in a neutral position were also excluded.

### 2.1. Dissection

The ankle specimens were stabilized in a lateral position with the ankle in neutral position. The lateral side of the ankle was completely exposed using detailed dissection to remove skin and soft tissue overlying the lateral hind foot. Care was taken to avoid injury or disruption to the native anatomy.

**Figure 1:**
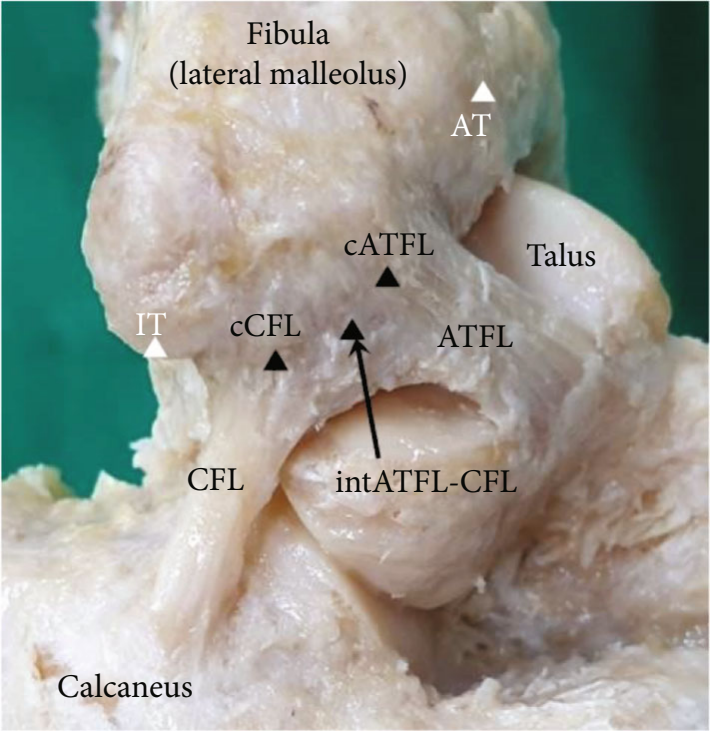
Lateral view of a right ankle in neutral plantar flexion and dorsiflexion, demonstrating the anatomic footprint sites of the lateral ankle ligaments and bony landmarks. AT: the articular tip of the lateral malleolus; ATFL: anterior talofibular ligament; cATFL: the center of the fibular footprint of the ATFL; cCFL: the center of the fibular footprint of the CFL; CFL: calcaneofibular ligament; intATFL-CFL: the intersection point of the fibular origin of the ATFL and CFL; IT: the inferior tip of the lateral malleolus.

After careful dissection, the number of ATFL bundles was noted to detect anatomical variability. Next, the anterior border of distal fibula (lateral malleolus) was confirmed using combined visual inspection and direct palpation of bone with all soft tissues removed. The following two reference points were marked: the articular tip of the lateral malleolus (AT) and the inferior tip of the lateral malleolus (IT). Further dissection to identify the center of the fibular footprint of the ATFL and CFL was performed (cATFL and cCFL, respectively). Finally, the intersection point of the fibular origin of the ATFL and CFL (intATFL-CFL) was identified by minimal dissection of the most inferior and posterior fibers of the ATFL and the most anterior fiber of the CFL (Figure 1). The points identified for this study were defined as follows.

1. The articular tip of the lateral malleolus (AT) was defined as the anterior fibular tubercle located most superior to the anterior border of the lateral malleolus
2. The inferior tip of the lateral malleolus (IT) was defined as the tip located most inferior to the anterior border of the lateral malleolus
3. The center of the fibular footprint of the ATFL (cATFL) was defined as the midcentral point bisecting the superior and inferior margins of the ATFL band at the fibular attachment
4. The center of the fibular footprint of the CFL (cCFL) was defined as the midcentral point bisecting the superior and inferior margins of the CFL band at the fibular attachment
5. The intersection point of the fibular origin of the ATFL and CFL (intATFL-CFL) was defined as the point where the most inferior and posterior fiber of the ATFL band and the most superior and anterior fiber of the CFL band intersect each other

### 2.2. Measurements

If the ATFL had multiple bundles, the center of the footprint including all bundles was used for measurements. The distances from the center of the fibular footprint of the ATFL and CFL to the inferior tip of the lateral malleolus were measured using a flexible surgical ruler. Also, the distance from the intersection point of the fibular origin of the ATFL and CFL to the inferior tip of the lateral malleolus and the distance from the articular and inferior tips of the lateral malleolus were measured. The identification of landmarks and measurements of the distances were performed independently by two researchers. Each independent researcher repeatedly measured the distance twice after identifying the landmarks. The averages of the two researchers' measurements were adopted as data for each specimen.

### 2.3. Statistical Analysis

Inter- and intraobserver reliabilities for all measurements were calculated by the intraclass correlation coefficient (ICC). According to the definition of Landis and Koch [21], ICCs of 0.81 to 1.00, 0.61 to 0.80, 0.41 to 0.60, 0.21 to 0.40, and 0.00 to 0.20 were interpreted as excellent, good, moderate, fair, and poor, respectively. Each measurement was presented using the mean, standard deviation, and range; since the number of specimens was more than 30, it was applicable to the normality assumption. For comparison between the single-bundle ATFL and double-bundle ATFL groups, a two-sample *t*-test was used. All statistical analyses were performed using SPSS 25.0 software (SPSS, Chicago, IL), and a *p* value less than 0.05 was considered statistically significant.

## 3. Results

Intraclass correlation coefficients were generated for all measurements. All measurements were higher than 0.8 (indicating acceptable reliability) and were employed in the study.

The distance (mean ± standard deviation) between the center of the fibular footprint of the ATFL and inferior tip of the lateral malleolus was 15.9 ± 3.2 mm. The distance between the center of the fibular footprint of the CFL and inferior tip of the lateral malleolus was 8.6 ± 2.9 mm. The distance between the intersection point of the fibular origin of the ATFL and CFL and inferior tip of the lateral malleolus was 12.0 ± 2.5 mm (Table 1). The distance between the AT and IT and the distance between cATFL and IT were significantly greater in males than in females (Table 2).

**Figure 2:**
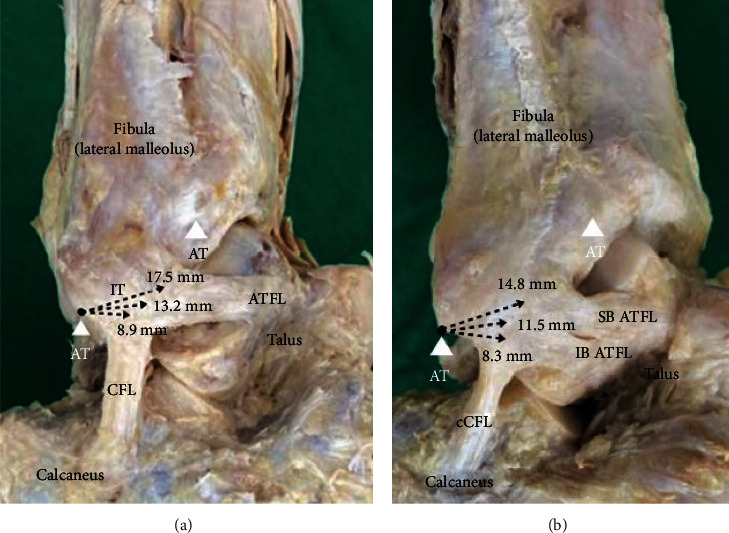
Lateral view of a right ankle depicting distances from the inferior tip of the lateral malleolus to the anatomic footprint sites of the lateral ankle ligaments. The bundle type of the anterior talofibular ligament was classified into a single type with an isolated band (a) and a double type with a divided superior band and inferior band (b). AT: the articular tip of the lateral malleolus; ATFL: anterior talofibular ligament; CFL: calcaneofibular ligament; IB ATFL: inferior band ATFL; IT: the inferior tip of the lateral malleolus; SB ATFL: superior band ATFL.

Of the 105 specimens, 42 (40%) specimens had single-bundle ATFL (type 1) and 63 (60%) specimens had a double-bundle pattern (type 2). There were no triple-bundle pattern specimens (Figure 2). With regard to the type of the bundle pattern of ATFL, there were statistically significant differences in the distance from cATFL to IT between the two groups (*p* < 0.001). The average distance from cATFL to IT was 17.5 ± 3.2 mm in type 1 and 14.8 ± 2.7 mm in type 2. However, there was no significant difference between the two groups in the distance from cCFL to IT (type 1: 8.9 ± 3.2, type 2: 8.3 ± 2.7, *p* = 0.266). In terms of the distance from the intersection point of the fibular origin of the ATFL and CFL to IT, significant differences were observed between the two groups (type 1: 13.2 ± 2.6, type 2: 11.5 ± 2.2, *p* = 0.001).

In addition, the three distances from the inferior tip of the lateral malleolus to anatomic footprint sites of the lateral ankle ligaments were calculated as a ratio of the length between the articular tip and inferior tip of the lateral malleolus. All ratio values are listed in Table 3. With regard to the type of the bundle pattern of ATFL, there were statistically significant differences in the ratio of cATFL and intATFL-CFL between the two groups. Further, all ratio values between the males and females showed no statistically significant differences (Table 4).

## 4. Discussion

A clear understanding of the anatomical location of the ligaments in relation to the bony landmark is important for surgeons performing anatomic reconstruction of the lateral ankle ligaments. The first contribution of the present study is to propose that surgeons can use a reference ratio to locate the fibular tunnel for anatomic reconstruction of the lateral ankle ligament, particularly in a patient who is much smaller or larger than average. The second is that there is a difference in the location of the fibular tunnel for anatomic reconstruction of the lateral ankle ligament between the single and double fascicular ATFL.

Most ankle stabilization surgeries involve repairing or reconstructing the ATFL and/or CFL. Numerous surgical procedures for chronic ankle instability have reported good clinical results [10]; but in order to overcome shortcomings such as wound complications in the open technique, minimally invasive surgery techniques have been used recently [17]. Minimally invasive surgery (MIS) for CAI includes anatomical repairs and reconstruction using arthroscopic [15, 19] and percutaneous techniques [7]. These MIS techniques commonly use bone anchors or construct bone tunnels at the anatomical origin and insert ATFL and CFL without open exposure. In particular, when both ATFL and CFL are reconstructed, a single fibular tunnel is required to insert the fibular stem by converting the ATFL and CFL into an anatomical Y graft [7, 8, 19, 20]. Therefore, for a more anatomically correct reconstruction, it is necessary to understand the anatomy of the common origin of ATFL and CFL in more detail.

The previous cadaveric study by Matsui et al. [8] suggested the fibular obscure tubercle (FOT) as a bony landmark for identifying the fibular footprint of the lateral ankle ligament. Some authors described the articular and inferior tips of the fibula (lateral malleolus) as a reference point [22–24]. However, it is somewhat insufficient to determine the location of the fibular tunnel as a common origin site of the ATFL and CFL with only one or two reference points in MIS procedures for CAI. This is because the distance between the bony landmark of the fibula and fibular footprint of the ATFL and CFL can be measured differently depending on the size and race of the cadaver. In previous cadaveric studies using 60 and 152 cadavers [23, 25], the ATFL and CFL were found to connect to each other at the anterior border of the lateral malleolus. The results of this study also showed connective fibers between ATFL and CFL covering the surface layer of the inferior part of ATFL and the anterior part of CFL in all 105 specimens. Moreover, topographically, both ATFL and CFL origins have a single confluent footprint on the anterior border of the distal fibula [26]. Therefore, the location of the single fibular tunnel for the common origin of ATFL and CFL is considered to be most anatomically reasonable to be the intersection point of the fibular origin of the ATFL and CFL (intATFL-CFL).

The intATFL-CFL was previously identified at the fibular obscure tubercle (FOT) by Buzzi et al. [27]. Based on this reference, it is recommended as an anatomical landmark for the location of the fibular tunnel when reconstructing the lateral ankle ligament [7]. However, a recent cadaveric study [8] has shown that the FOT is located proximally close to the intATFL-CFL and not at the same location. It was also found that FOT could not be manually detected in all patients. According to research on MIS techniques, if the FOT is not detectable with fluoroscopic view imaging or palpation, the inferior one-third point between the articular tip (AT) and the inferior tip (IT) of the lateral malleolus on its anterior border is suggested as an alternative, but no evidence for such an alternative was found.

**Figure 3:**
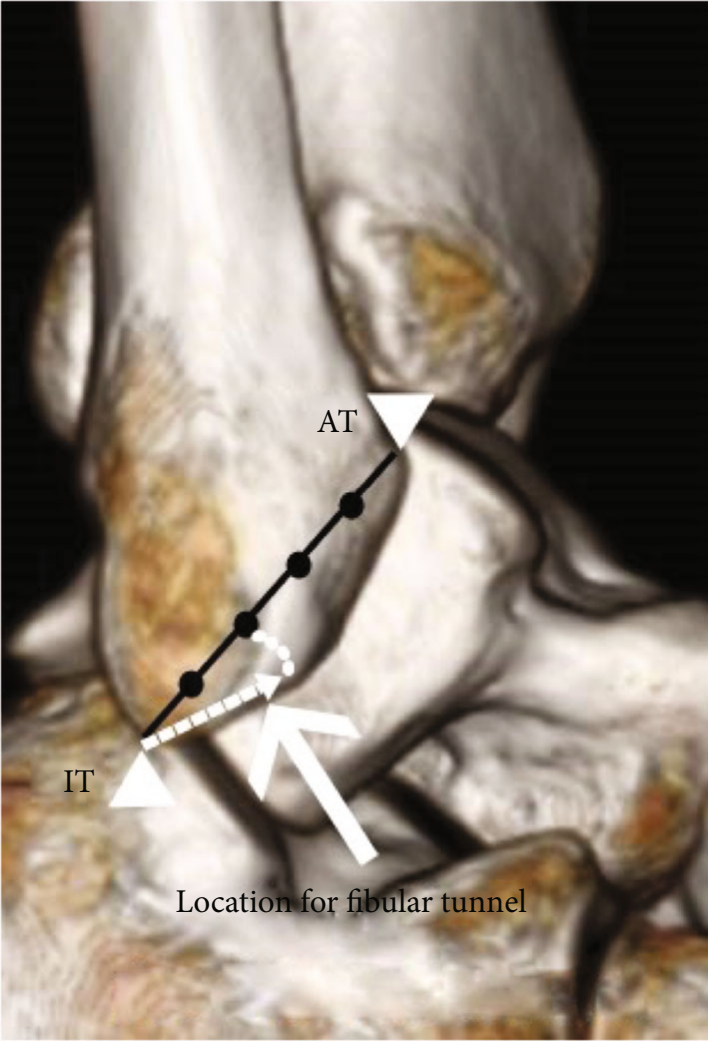
Schematic diagram with three-dimensional CT reconstruction of the lateral side of a normal hind foot. The black circles represent points that divide the distance from AT to IT into 5 equal parts. The white dotted line shows the extension area of the inferior 40% of the distance from AT to IT. The white arrow indicates the location of the fibular tunnel for clinical application. AT: articular tip of the lateral malleolus; IT: inferior tip of the lateral malleolus.

A systematic review showed that the origins of the ATFL and CFL were located around 10-14 and 5-8 mm from the IT, respectively [17]. On the other hand, the fibular footprint of the ATFL and CFL was located 15.9 ± 3.2 and 8.6 ± 2.9 mm in our study. These differences in values were not significant and may be accounted for by the size and race of the cadavers and the anatomic variability. Further, the results of our study show that there is a difference in the absolute distance due to the difference in body size between males and females; however, the ratio did not differ by sex. We, therefore, determined that it would be more reliable and clinically useful to describe these values in the ratio rather than the absolute distance. In the present study, the ratio of the intATFL-CFL location based on the distance along the anterior fibular border for all cadavers was nearly 0.4. Using this reference ratio, dividing the distance from AT to IT into 5 equal parts and making the fibular tunnel along the extension of the just inferior 40% point are believed to be a more anatomically correct reconstruction of the lateral ankle ligament compared to using the current surgical techniques (Figure 3).

Although ligament injury combined with bony avulsion fracture may interfere with the reference using the bony landmarks, the importance of the applicability of this ratio using surgically relevant bony landmarks for reference during the anatomical reconstruction procedure cannot be underestimated. The indication for performing an anatomical reconstruction procedure is instability from chronic rupture rather than acute rupture of the lateral ligament. A sufficient incision may be made to expose the ligament attachment, or an arthroscopy may be used to navigate the ligament attachment. However, in chronic ankle lateral instability (CALI), the surgeon is more likely to face a complex situation in which the native ligament centers are not visible due to severe ligament damage. Use of this ratio using bony landmarks facilitates consistent and anatomical placement. Additionally, it may clinically enable minimally invasive anatomical reconstruction of the lateral ligament. A previous study has mentioned that sufficient incision is the key to anatomic reconstruction of the ligaments, but it is emphasized that attention to wound complications and nerve damage is essential [28]. Positioning the tunnel location before surgery helps to minimize the incision and reduce the risk of wound complications and nerve damage through reduction of the surgical incision. Furthermore, additional establishing unnecessary portals for tunnels can be avoided during minimally invasive surgery using arthroscopy.

The ultimate purpose of ligament reconstruction is to recreate the course of the injured ligaments [29]. Thus, ligament reconstruction has been mainly used for failure of the previous ligament surgery, athletes who want to perform high-intensity activities, generalized laxity, and insufficient ligament tissue for direct repair [28, 30]. Unfortunately, nonanatomical ligament reconstruction has been reported to show restricted ankle joint motion and early arthritic changes compared to anatomical reconstruction. In contrast, anatomical ligament reconstruction has been reported to have good clinical outcomes after the surgical procedure [28, 29, 31–33]. It can be assumed that anatomical ligament reconstruction may better mimic native joint mechanics. For more anatomical reconstruction of ligament, various factors such as the type and strength of ligament to be reconstructed (autograft vs. allograft) and the method of drilling the tunnel and fixation method for reconstructed ligaments should be considered. Only positioning the accurate anatomical location of the tunnel cannot guarantee the best biomechanics, but we believe that the results of our study could lead to more accurate anatomical reconstruction of ligament and may contribute to improving both basic and clinical outcomes after surgery.

Previous studies reported anatomical variations of the ATFL with regard to the number of bundles [18, 22, 23, 25]. Thus, there is a possibility that the location of the fibular tunnel may differ depending on the type of the bundle pattern of the ATFL. ATFL could be classified into 3 bundle types (single, double, or multiple). The single-band type consists of an isolated band. The double-band type is divided by superior and inferior fibers. The multiple-band type is divided by triple bands or more. In a systemic review [17], the frequency of the bundle pattern was reviewed in a total of 263 specimens from 10 previous studies. This review showed that the incidences of single-, double-, and triple-bundle ATFL were 162 (61.6%), 94 (35.7%), and 7 (2.7%), respectively. However, our study showed different results. Of the 105 specimens, 42 (40%) had single-bundle ATFL (type 1) and 63 (60%) had a double-bundle pattern (type 2). There were no specimens of the triple-bundle pattern. Moreover, recent studies with larger sample sizes in Japanese ankles showed that the frequency of the ATFL bundle patterns was similar to our results. Edama et al. [34] studied 81 ankles and found that type 2 occurred most frequently (57%). Kobayashi et al. [25] studied 152 ankles and found that type 2 was the most frequent (54.6%) as well. This uncertainty may be attributable to differences in human races or bias based on the small number of specimens.

Although no conclusions were reached regarding frequencies of ATFL bundle patterns, there may be a possibility that the location of the fibular tunnel may differ depending on the type of ATFL bundle patterns. To date, there is no description of a comparative analysis of intATFL-CFL according to the number of ATFL bundles in the literature. Although the measured value was the difference in small units such as mm, our findings have shown that both the absolute value and the ratio of the location of intATFL-CFL differ significantly according to the number of ATFL bundles. In the double fascicular ATFL, the fibular tunnel for more anatomically correct ligament reconstruction would have to be located more distal than that in single fascicular ATFL, although achieving this small difference may not be surgically feasible.

This study was limited by the use of fixed cadavers to evaluate the morphological characteristics of the lateral ankle ligament. With regard to postmortem changes, there may be differences in the measurements taken for a live person and those from a cadaver. Further, the cadavers were limited to those of elderly individuals (mean age, 76.4 years) and the trauma history of the ankle may not be fully certain because the corpse specimens donated for research do not provide the past medical history.

## 5. Conclusions

The present study suggests a reference ratio that can help surgeons to locate the fibular tunnel for more anatomically correct reconstruction of the lateral ankle ligament. Also, it may be necessary to make a difference in the location of the fibular tunnel according to the number of ATFL bundles during surgery. Further clinical trials on this will be needed in the future.

## Figures and Tables

**Table 1:** The distances between the fibular footprint of the lateral ankle ligaments and the selected bony landmarks (*n* = 105 specimens).

Measurement point	Distance (mm)			
	Mean	SD	Minimum	Maximum
cATFL~IT	15.9	3.2	10	25
cCFL~IT	8.6	2.9	0	16
intATFL-CFL~IT	12.0	2.5	5.5	19.5
AT~IT	31.8	2.6	25.5	40

AT: the articular tip of the lateral malleolus; ATFL: anterior talofibular ligament; cATFL: the center of the fibular footprint of the ATFL; cCFL: the center of the fibular footprint of the CFL; CFL: calcaneofibular ligament; intATFL-CFL: the intersection point of the fibular origin of the ATFL and CFL; IT: the inferior tip of the lateral malleolus; SD: standard deviation.

**Table 2:** The distances between the fibular footprint of the lateral ankle ligaments and the selected bony landmarks by sex.

Measured distance (mm)	Males (*n* = 65)	Females (*n* = 40)	*p* value
cATFL~IT	16.5 ± 3.3	14.7 ± 2.5	0.003
cCFL~IT	8.5 ± 3.1	8.6 ± 2.5	0.810
intATFL-CFL~IT	12.5 ± 2.7	11.6 ± 2.1	0.093
AT~IT	32.6 ± 2.3	30.1 ± 2.3	<0.001

Data are mean ± standard deviation. AT: the articular tip of the lateral malleolus; ATFL: anterior talofibular ligament; cATFL: the center of the fibular footprint of the ATFL; cCFL: the center of the fibular footprint of the CFL; CFL: calcaneofibular ligament; intATFL-CFL: the intersection point of the fibular origin of the ATFL and CFL; IT: the inferior tip of the lateral malleolus; SD: standard deviation.

**Table 3:** The ratio of three distances from the inferior tip of the lateral malleolus to the anatomic footprint of the lateral ankle ligaments to the length between the articular tip of the lateral malleolus and inferior tip of the lateral malleolus by the type of the bundle pattern of ATFL (*n* = 105 specimens).

Measurements	Ratio			
	Total	Type 1 (*n* = 42)	Type 2 (*n* = 63)	*p* value
(cATFL-IT)/(AT-IT)	0.500 ± 0.091	0.544 ± 0.096	0.472 ± 0.076	<0.001
(cCFL-IT)/(AT-IT)	0.267 ± 0.084	0.276 ± 0.089	0.265 ± 0.081	0.504
(intATFL-CFL)/(AT-IT)	0.386 ± 0.070	0.410 ± 0.074	0.368 ± 0.063	0.003

Data are mean ± standard deviation. AT: the articular tip of the lateral malleolus; ATFL: anterior talofibular ligament; cATFL: the center of the fibular footprint of the ATFL; cCFL: the center of the fibular footprint of the CFL; CFL: calcaneofibular ligament; intATFL-CFL: the intersection point of the fibular origin of the ATFL and CFL; IT: the inferior tip of the lateral malleolus.

**Table 4:** The ratio of three distances from the inferior tip of the lateral malleolus to the anatomic footprint of the lateral ankle ligaments to the length between the articular tip of the lateral malleolus and inferior tip of the lateral malleolus by sex.

Measurements	Ratio		
	Males (*n* = 65)	Females (*n* = 40)	*p* value
(cATFL-IT)/(AT-IT)	0.509 ± 0.099	0.488 ± 0.077	0.264
(cCFL-IT)/(AT-IT)	0.260 ± 0.092	0.284 ± 0.070	0.177
(intATFL-CFL)/(AT-IT)	0.385 ± 0.078	0.386 ± 0.056	0.933

Data are mean ± standard deviation. AT: the articular tip of the lateral malleolus; ATFL: anterior talofibular ligament; cATFL: the center of the fibular footprint of the ATFL; cCFL: the center of the fibular footprint of the CFL; CFL: calcaneofibular ligament; intATFL-CFL: the intersection point of the fibular origin of the ATFL and CFL; IT: the inferior tip of the lateral malleolus.

## Data Availability

The data used to support the findings of this study are available from the corresponding author upon request.

## References

[1] Balduini F. C., Tetzlaff J. (1982). Historical perspectives on injuries of the ligaments of the ankle. *Clinics in Sports Medicine*.

[2] Fong D. T.-P., Hong Y., Chan L.-K., Yung P. S.-H., Chan K.-M. (2007). A systematic review on ankle injury and ankle sprain in sports. *Sports Medicine*.

[3] Freeman M. A. (1965). Instability of the foot after injuries to the lateral ligament of the ankle. *Journal of Bone and Joint Surgery. British Volume (London)*.

[4] Kumai T., Takakura Y., Rufai A., Milz S., Benjamin M. (2002). The functional anatomy of the human anterior talofibular ligament in relation to ankle sprains. *Journal of Anatomy*.

[5] Sugimoto K., Samoto N., Takaoka T., Takakura Y., Tamai S. (1998). Subtalar arthrography in acute injuries of the calcaneofibular ligament. *Journal of Bone and Joint Surgery. British Volume (London)*.

[6] van den Bekerom M. P. J., Oostra R. J., Alvarez P. G., van Dijk C. N. (2008). The anatomy in relation to injury of the lateral collateral ligaments of the ankle: a current concepts review. *Clinical Anatomy*.

[7] Glazebrook M., Eid M., Alhadhoud M., Stone J., Matsui K., Takao M. (2018). Percutaneous ankle reconstruction of lateral ligaments. *Foot and Ankle Clinics*.

[8] Matsui K., ESSKA AFAS Ankle Instability Group, Oliva X. M. (2017). Bony landmarks available for minimally invasive lateral ankle stabilization surgery: a cadaveric anatomical study. *Knee Surgery, Sports Traumatology, Arthroscopy*.

[9] Takao M., Miyamoto W., Matsui K., Sasahara J., Matsushita T. (2012). Functional treatment after surgical repair for acute lateral ligament disruption of the ankle in athletes. *The American Journal of Sports Medicine*.

[10] Guillo S., Bauer T., Lee J. W. (2013). Consensus in chronic ankle instability: aetiology, assessment, surgical indications and place for arthroscopy. *Orthopaedics & Traumatology, Surgery & Research*.

[11] Lynch S. A., Renström P. A. F. H. (1999). Treatment of acute lateral ankle ligament rupture in the athlete. Conservative versus surgical treatment. *Sports Medicine*.

[12] Petersen W., Rembitzki I. V., Koppenburg A. G. (2013). Treatment of acute ankle ligament injuries: a systematic review. *Archives of Orthopaedic and Trauma Surgery*.

[13] Strauss J. E., Forsberg J. A., Lippert F. G. (2007). Chronic lateral ankle instability and associated conditions: a rationale for treatment. *Foot & Ankle International*.

[14] White W. J., McCollum G. A., Calder J. D. (2016). Return to sport following acute lateral ligament repair of the ankle in professional athletes. *Knee Surgery, Sports Traumatology, Arthroscopy*.

[15] Brown A. J., Shimozono Y., Hurley E. T., Kennedy J. G. (2020). Arthroscopic versus open repair of lateral ankle ligament for chronic lateral ankle instability: a meta-analysis. *Knee Surgery, Sports Traumatology, Arthroscopy*.

[16] Porter D. A., Kamman K. A. (2018). Chronic lateral ankle instability: open surgical management. *Foot and Ankle Clinics*.

[17] Matsui K., ESSKA AFAS Ankle Instability Group, Burgesson B. (2016). Minimally invasive surgical treatment for chronic ankle instability: a systematic review. *Knee Surgery, Sports Traumatology, Arthroscopy*.

[18] Szaro P., Ghali Gataa K., Polaczek M., Ciszek B. (2020). The double fascicular variations of the anterior talofibular ligament and the calcaneofibular ligament correlate with interconnections between lateral ankle structures revealed on magnetic resonance imaging. *Scientific Reports*.

[19] Guillo S., Archbold P., Perera A., Bauer T., Sonnery-Cottet B. (2014). Arthroscopic anatomic reconstruction of the lateral ligaments of the ankle with gracilis autograft. *Arthroscopy Techniques*.

[20] Guillo S., Cordier G., Sonnery-Cottet B., Bauer T. (2014). Anatomical reconstruction of the anterior talofibular and calcaneofibular ligaments with an all-arthroscopic surgical technique. *Orthopaedics & Traumatology, Surgery & Research*.

[21] Landis J. R., Koch G. G. (1977). The measurement of observer agreement for categorical data. *Biometrics*.

[22] Clanton T. O., Campbell K. J., Wilson K. J. (2014). Qualitative and quantitative anatomic investigation of the lateral ankle ligaments for surgical reconstruction procedures. *Journal of Bone and Joint Surgery*.

[23] Kakegawa A., Mori Y., Tsuchiya A., Sumitomo N., Fukushima N., Moriizumi T. (2019). Independent attachment of lateral ankle ligaments: anterior talofibular and calcaneofibular ligaments - a cadaveric study. *The Journal of Foot and Ankle Surgery*.

[24] Taser F., Shafiq Q., Ebraheim N. A. (2006). Anatomy of lateral ankle ligaments and their relationship to bony landmarks. *Surgical and Radiologic Anatomy*.

[25] Kobayashi T., Suzuki D., Kondo Y. (2020). Morphological characteristics of the lateral ankle ligament complex. *Surgical and Radiologic Anatomy*.

[26] Neuschwander T. B., Indresano A. A., Hughes T. H., Smith B. W. (2013). Footprint of the lateral ligament complex of the ankle. *Foot & Ankle International*.

[27] Buzzi R., Brenner E., Segoni F., Inderster A., Aglietti P. (1993). Reconstruction of the lateral ligaments of the ankle: an anatomic study with evaluation of isometry. *Journal of Sports Traumatology and Related Research*.

[28] Lee D. W., Park I. K., Kim M. J. (2019). Three-dimensional computed tomography tunnel assessment of allograft anatomic reconstruction in chronic ankle instability: 33 cases. *Orthopaedics & Traumatology, Surgery & Research*.

[29] Tourne Y., Mabit C. (2017). Lateral ligament reconstruction procedures for the ankle. *Orthopaedics & Traumatology, Surgery & Research*.

[30] Dierckman B. D., Ferkel R. D. (2015). Anatomic reconstruction with a semitendinosus allograft for chronic lateral ankle instability. *The American Journal of Sports Medicine*.

[31] Jung H. G., Kim T. H., Park J. Y., Bae E. J. (2012). Anatomic reconstruction of the anterior talofibular and calcaneofibular ligaments using a semitendinosus tendon allograft and interference screws. *Knee Surgery, Sports Traumatology, Arthroscopy*.

[32] Krips R., van Dijk C. N., Halasi T. (2001). Long-term outcome of anatomical reconstruction versus tenodesis for the treatment of chronic anterolateral instability of the ankle joint: a multicenter study. *Foot & Ankle International*.

[33] Schmidt R., Cordier E., Bertsch C. (2004). Reconstruction of the lateral ligaments: do the anatomical procedures restore physiologic ankle kinematics?. *Foot & Ankle International*.

[34] Edama M., Kageyama I., Kikumoto T. (2018). Morphological features of the anterior talofibular ligament by the number of fiber bundles. *Annals of Anatomy*.

